# Public, private and personal: Qualitative research on policymakers' opinions on smokefree interventions to protect children in 'private' spaces

**DOI:** 10.1186/1471-2458-10-797

**Published:** 2010-12-31

**Authors:** Gareth Rouch, George Thomson, Nick Wilson, Sheena Hudson, Richard Edwards, Heather Gifford, Tolotea Lanumata

**Affiliations:** 1 University of Otago, Wellington; Box 7343 Wellington, New Zealand; 2 Whakauae Research Services; Box 102 Whanganui, New Zealand

## Abstract

**Background:**

Governments use law to constrain aspects of private activities for purposes of protecting health and social wellbeing. Policymakers have a range of perceptions and beliefs about what is public or private. An understanding of the possible drivers of policymaker decisions about where government can or should intervene for health is important, as one way to better guide appropriate policy formation. Our aim was to identify obstacles to, and opportunities for, government smokefree regulation of private and public spaces to protect children. In particular, to seek policymaker opinions on the regulation of smoking in homes, cars and public parks and playgrounds in a country with incomplete smokefree laws (New Zealand).

**Methods:**

Case study, using structured interviews to ask policymakers (62 politicians and senior officials) about their opinions on new smokefree legislation for public and private places. Supplementary data was obtained from the Factiva media database, on the views of New Zealand local authority councillors about policies for smokefree outdoor public places.

**Results:**

Overall, interviewees thought that government regulation of smoking in private places was impractical and unwise. However, there were some differences on what *was *defined as 'private', particularly for cars. Even in public parks, smoking was seen by some as a 'personal' decision, and unlikely to be amenable to regulation. Most participants believed that educative, supportive and community-based measures were better and more practical means of reducing smoking in private places, compared to regulation.

**Conclusions:**

The constrained view of the role of regulation of smoking in public and private domains may be in keeping with current political discourse in New Zealand and similar Anglo-American countries. Policy and advocacy options to promote additional smokefree measures include providing a better voice for childrens' views, increasing information to policymakers about the harms to children from secondhand smoke and the example of adult smoking, and changing the culture for smoking around children.

## Background

### Privacy, public policy and health

In order to protect children, governments reach into both public and private settings. These actions include investigations into the risk of harm to children, and interventions to decrease or prevent such harm in the home [1,2]. The interventions can be seen as part of a spectrum of government intervention into, or regulation of, private life and activities for health protection purposes. The spectrum of intervention ranges from laws on reproduction,[3] abortion,[4] and suicide,[5] to compulsory food additives,[6] fluoridation of water supplies,[7] and environmental health protection (eg, reducing substance and product toxicity) [8]. Some governments also intervene in aspects of behaviour in private vehicles, where those behaviours threaten the welfare of children or others (eg, requirements for seat belts and child restraints,[9] and bans on cellphone use while driving in cars) [10].

Exactly what constitutes 'public' and 'private' is unclear [11]. The distinctions of public and private venues and activities 'bleed into one another',[12] with wide disagreement about their meanings and consequences.

Common themes in the distinctions between public and private include the degree to which spaces and activities are *physically *or *visually *open/closed to the public, or are open to the *concern *of the community and government agencies [13]. Thus some seemingly private *activities*, eg, the use of violence to children in a private space, are seen by some societies as an appropriate concern of the state. Some *spaces*, eg, private cars, can be considered to contain both activities of public concern (eg, driving without due care) and some personal or private activities (eg, eating, talking) [14].

### Smoking around children and health

There is no known safe level of exposure to tobacco smoke pollution (secondhand smoke - SHS),[15] and the exposure of children to SHS has adverse implications for their immediate and long-term health [16,17]. Seeing smoking by adults and peers may also increase childrens' risk of experimenting with, and becoming addicted to tobacco, due to the effects of the *example *of smoking [18,19].

Comprehensive tobacco control programmes, which reduce the prevalence of smoking, are the primary intervention of governments for reducing smoking around children [20]. Specific population-level policies may further reduce smoking around children. These could include smokefree schools, smokefree outdoor areas,[21] smokefree cars,[22] and media campaigns to change smokers' behaviours around children [23,24]. There is evidence that smokefree homes and knowledge about the SHS hazard may increase the adoption of smokefree cars,[25] and smokefree public places may increase the likelihood of smokefree homes [26].

The extent to which central governments and local authorities should go to protect children from smoking is contested [27-31]. In Australia 'the protection of vulnerable children ... [has been] a powerful and persuasive theme' in achieving smokefree car laws,[32] but most countries do not yet have such laws. The issues around introducing campaigns or legislating to promote or enforce smokefree homes are complex, including the legitimacy of intervening on behaviours in a 'private' space, and practical issues like the availability of alternate places for smoking [33,34]. Policymakers may therefore question the appropriateness of any government role in smokefree homes [35].

Previous research has indicated some of the influences upon policymakers' views on tobacco policy interventions. These include their ideology, and attitudes to the role of government [36,37]. Relevant policy research also includes investigations into beliefs about the role of government in particular areas,[35] and the nature of the rights involved [38].

Even where policymakers are inclined to favour interventions, there is the question of where such changes are in their priorities [35,39-42]. Change is easier to advance when tobacco control interventions are demonstrably effective and cost-effective, with simple and issue-free implementation,[43] and where change is seen to have political advantages [44]. When policymakers know of the success of interventions elsewhere, the barriers are lower [45-47].

To extend this research, in this article we use a case study (New Zealand policymakers) to explore policymakers' beliefs about what is public or private; and the degree to which governments should intervene to regulate smoking in various settings. Public/private considerations are relevant to a wide range of policy areas, and better understandings of the considerations may enable more informed policy decisions [48-50].

### The New Zealand context

New Zealand national law requires that nearly all indoor workplaces and public places, and all school grounds are smokefree [51,52]. Local authorities have very limited powers to regulate behaviour in homes and cars, but could control smoking in some outside public places [53]. Over 30% of local authorities have non-enforceable 'educative' smokefree policies for at least council playgrounds [54]. There is no legislation about smoking in homes or 'non-work' cars, but government-funded social marketing campaigns (albeit of modest scale) have been used to encourage family-level adoption of smokefree home and car policies [55,56]. National surveys of smokers and non-smokers have found strong support for smokefree car and playground laws (over 80%) [57-59].

It was estimated in the year 2000 that the effects of SHS in New Zealand include 50 sudden infant deaths/year,[60] over 500 child hospital admissions, and thousands of episodes of childhood asthma and glue ear operations [61]. These effects were largely due to SHS exposure in homes and cars. However, research indicates that a number of New Zealand politicians during 2000-2005 were sceptical about the extent of harm of exposure to SHS [62].

### Aims

Our main aim in this qualitative case study was ascertain the range and nature of New Zealand policymaker opinions on the regulation of smoking in homes, cars and public parks and playgrounds. More specifically, we aimed to identify obstacles to, and opportunities for, government smokefree regulation of private and public spaces to protect children.

## Method

To investigate opinions, we used semi-structured in-depth interviews with New Zealand national and regional policymakers, coupled with a review of the published statements of councillors in New Zealand local authorities (councils). Interviews were chosen as the primary source, due to the need for nuance, and to allow the unexpected to be explored. For this research, policymakers were defined as national and regional politicians, senior non-elected officials in central government or District Health Boards (DHBs) and local councillors. The regional politicians were DHB board members. Ethics approval for the interviews was obtained from the Department of Public Health, University of Otago, Wellington.

### Purposive interview recruitment

Between April 2008 and February 2009 we approached 48 current and past Members of Parliament (MPs), five current DHB board members, and 54 current and past senior officials who were, or had been, in a position to influence health policy within the past five years (a total of 107). Almost all the officials had at least 10 years experience as officials, and most were or had been in a position to present verbal or written evidence to Cabinet Ministers. To ensure participation from across the political spectrum, we approached 20 National Party and 14 Labour Party MPs (the two major political parties) as well as 14 MPs from other parties. To help recruitment, the anonymity of the interview material was repeatedly stressed. An information sheet on the interview process was supplied to interviewees, and a consent form signed by them before the interview.

### Interview process

A standard semi-structured interview format was used. Subsidiary prompts ensured that all relevant issues could be addressed. Interview questions explored the necessity, practicality, and value of smokefree regulations or other interventions for parks, playgrounds, cars and homes (see Table 1). We were particularly interested in the feelings, beliefs, and ideologies underlying the opinions expressed. We did not provide any information to interviewees, eg, on public support for regulating smokefree cars and playgrounds. Nor did we challenge opinions or claims. We did not present any particular position, as we felt that could influence the responses.

**Table 1:** Relevant* interview guide questions

1) Your professional background
• How long have you been involved in public policy processes?

• Can you tell me about what that role involved?

2) Your opinion of the role of the government in health policies

• What should be the government's role in promoting healthy living?

3) Your views on adult smoking in general (both in the presence of children under 16 and elsewhere)

• What are your ideas on the relative rights of adults and children under 16, in regard to smoking around children?

4) Your ideas on some smokefree policies

• In general what sort of places that are not smokefree in NZ do you think should be smokefree?

• Do you know of anywhere where smoking in these types of places such as cars, parks or playgrounds or streets has been banned?

• IF NOT ALREADY COVERED. What is your view on policies that would ban smoking in: cars, parks and playgrounds, or streets around shopping areas, where there are children under 16?

5) A few final questions

• Where would you place yourself in a left-right ideology scale: Far left, left, centre left, centre, centre right, right or far right?

• Generally, do you think the level of government regulation of the private sector is too little, too much, or about right?

• What has been your personal experience of smoking and its effects?

• Is there anything else you would like to add, or issues you think we should cover?

• Are there other people you think we should talk to, or documents we should look at for this study?

* Only questions relevant to this article are given here

We conducted 62 interviews, of which 22 were national or regional politicians (MPs or DHB board members) and 40 were officials. There was a higher response rate among officials (74%) compared to MPs (35%). MPs gave a range of reasons for declining interviews, including time, the October 2008 election, and 'other priorities'. Of the MPs, nine were from 'left/center-left' parties (Greens and Labour), four were from 'centre' parties (New Zealand First, United Future and the Māori Party) and four from the 'right' (National Party). Because we had a sufficient range of politicians and officials, and because we found repeated themes across the 'left-right' spectrum, we did not recruit further.

The interviews took up to 45 minutes, and were audio recorded. With the exception of four telephone interviews, all interviews were face-to-face. One interview was of three officials together, and another of two together.

### Media coverage of councillors

We were also interested in the opinions of local councillors. The spectrum of possible and relevant government interventions includes policies for smokefree outdoor places. Many New Zealand local authorities have and are continuing to introduce relevant policy measures, such as smokefree parks and playgrounds so we focused on this area of activity. To supplement the interview data, a Factiva media database search was conducted for the New Zealand 'region'. We searched for all New Zealand articles, for the period January 1998 - June 2008, which referred to the views of local authority councillors on smokefree policies. The search words used included combinations of 'smoking', 'smokefree', 'ban', 'council', 'councillor', 'parks', and/or 'playgrounds'. Fifteen councillor statements regarding smokefree park and playgrounds policies were found, all during 2005-2008.

### Data analysis

Interviews were transcribed, and read by at least two of the authors. Initially data from the interviews and media coverage were allocated to groups according to the pre-defined settings (eg, home, car, parks). Data was then coded in relation to the perception of privacy and views on regulation. Themes were modified as further themes emerged [63].

Data in each group were then examined for the key qualifications, reservations, nuances and ideological stances which interviewees used when they considered the implications of government regulation. The themes were discussed and re-checked against the data by at least two of the authors in order to agree revisions to major themes. A selection of the relevant data were then read by other co-authors to provide checks on the interpretation of the data.

## Results

We found complex reactions to the idea of smokefree regulation, from both politicians and officials, and from politicians on both the 'left' and 'right' of the political spectrum. This complexity was partly due to differing perceptions of what could be seen as public or private activities or spaces, and what behaviours were viewed as *personal *decisions, even in public spaces.

Policymakers generally perceived smoking in different *spaces *(homes, cars, and parks) as having different levels of privacy, and thus varying potential for regulation. Within each space, there were a range of opinions about smokefree interventions. The different perceptions appeared to stem partly from beliefs about the role of government and the nature of society. Interviewees generally believed that, for a variety of reasons, government could not, or should not intervene in 'private' spaces to regulate smoking.

### Homes

The home was considered by all interviewees as the venue most impervious to government smokefree regulation. Interviewees often approached the issue of intervention in the home as a question:

*'The rights of children [not to be exposed to smoke] perhaps are even greater [than adults], in theory. In practice, ... we then start getting into the situations of homes, ... how far can you go ... for the state, for example, to intervene to stop adults smoking around children*?'

Some weighed up a smoker's 'choice' against the wellbeing of children:

'In the home, ... it's a balance between the rights of the families to freedom and personal choice, but also the welfare of the children.'

Most interviewees deferred to a smokers' right to smoke, rather than the protection of children from SHS:

*'[Interventions on] smoking in our homes. Now that ... cuts across ... "what happens in my house is my [business]" ... my house, my castle. ... You're ... asking people to behave for a public good in a private space. ... The big barrier is that it's a step into private space*.'

But one interviewee suggested testing the boundaries:

'We should perhaps be a bit braver than we have been ...and [be] talking about the homes. I think that we should start to go there. ... the argument is you can't interfere with the sanctity of the home. In fact we interfere in the home the whole time ...the classic example was you couldn't convict a man of rape in his own home and that now is so completely unacceptable. ... we do change our views to what is acceptable at home.'

### Cars

The home and the car were considered by some to be similarly private spaces:

*'When we did the smokefree *[public and workplace] *legislation ... a man's home was his castle and you didn't stray there, and a car was sort of put into that same category.'*

Many viewed cars in two distinct ways. Private vehicles on roads were generally seen by interviewees as part of a public activity, *for traffic safety purposes*. The public were seen by them as receptive, or at least not resistant to, regulations which bear upon the *operation *of a car as a public activity. However, activities *inside *of vehicles were seen by many interviewees as occurring in private places, at least for smoking, if not for other activities. One said:

'I'm happy for police time to be used on policing seat belts. I'm less happy for police time to be used on policing smoking.'

Some saw the boundaries as changing with time:

*People's views on where the government could intervene on seat belts, for example, has shifted, over my lifetime. People's views about government intervening on tobacco has certainly shifted*.'

And others thought that the public might accept smokefree regulations for cars, at least when children are present:

*'There's already an expectation ... that certain activities in cars aren't acceptable, ... drinking, and driving crazy, and not wearing a seat belt, ... and ... people can see the argument ... for poor little kids trapped in this car, with parents smoking*.'

### Objections to, and support for, regulating for smokefree private spaces

Policymakers identified two main types of reasons why the regulation of private spaces would be ineffective or inappropriate; practical and ideological (although there was some overlap). The support given *for *the regulation of private spaces was largely based on the perceived need and duty to protect children. The central practical objection was the difficulty of enforcement, particularly in the home, but also in cars:

'I think some of that's very difficult to police, and I don't believe in having laws that you can't actually enforce in any way.'

Few interviewees were aware of the implementation of smokefree car laws in states and provinces of Australia, the USA and Canada. A further practicality (partly because of ideology) was that significant tobacco control legislation was:

*'Very difficult and expensive, if one uses that phrase in terms of political costs ... the best solutions with tobacco are legislatively impossible, even though they would actually achieve 99% of the [desired] outcome*.'

That political impracticality was because of the perceived strong public concern about the 'nanny state'. This meant that when interventions were considered:

'You've got to look at the broader context of what's happening, or else you run into the nanny state problem. ... there's a level of tolerance by society, in terms of state interference, [and] that level of tolerance is partly related to what other activities [are happening], and also partly related to the level of risk. And also, who that risks. ... So ... tactically, you have to be aware of what debates are running ... the public health community has to be strategic about what thing it's going to push for, [and] when.'

Some officials were very insistent on this point:

'It's very important [to remember that] ... what's useful, what's effective, what's practicable, what's politically acceptable, are all different things. ... it's what's going to be politically acceptable that ... we, in the bureaucracy at least, need to take account of. So we're not going to have radicals' programmes.'

### Ideology

Policymakers generally agreed that the ideological objections to smokefree regulation of private places were more trenchant than the practical ones. They thought that public discussion could quickly move away from the activity and place of smoking, and onto questions about what right a government has to regulate personal behaviour in private spaces:

*'The question of whether adults have a right to smoke at the cost of their children's health is a political question, in that sense. It depends how far the state goes to regulate individuals' liberties and behaviour*.'

A number of interviewees believed that possible smokefree legislation for the private home and car was not only impossible to enact, but *should *not be considered:

'Their car is their own property, their house is their own property. If they want to smoke [there] it's up to the individual.'

### Public parks and children's playgrounds

Interviewees' comments on smokefree policies for public parks contrasted strongly with their views on policies for private homes and cars, lacking the reservations and anxieties they expressed when considering the home. There was also much less concern for a possible public backlash.

Nevertheless, some interviewees believed that such polices still impacted upon a behaviour which was a personal choice, albeit one conducted in a public space. Because smoking was seen to be 'personal', many policy makers considered legislation to be an overly draconian means of decreasing smoking role-modelling to children in parks and playgrounds. The most appropriate measures, a number stated, were the voluntary smokefree policies developed and applied by local councils in New Zealand:

'At least a part of our communities ... would ... say "no, no, don't do that [regulate], it's ineffective". ... But [they would say]... "We want to protect our kids, we want to do this voluntarily".'

Interviewees who explored the idea of legislation thought it was best to first foster a cultural shift, one based at the community level. This might later be formalised by central government regulation.

'So it's bottom-up stuff, these are a lot more acceptable in communities than top-down. ... Do it voluntarily before you mandate it, ...you'd achieve it by having parents or community groups saying "this is what we want you to do to protect our kids".'

However, when we examined the published statements of New Zealand local authority councillors, some did not feel comfortable *even *with non-enforceable smokefree policies for parks. One councillor supported smokefree playgrounds,[64] but was reported as saying about a proposed smokefree parks policy:

*'There's too much of this social engineering thing going on in this country right now.'*[65]

Others said:

*'Total [park] bans reduced smokers to social lepers. It was still a legal pastime, and smokers should not be excluded from all public areas.'*[66]

*'People should have the freedom of choice to smoke outside.'*[67]

But over 25 councils (out of 73) had voted for some form of smokefree outdoors policy at the time of this study [54]. One mayor said, about a new council educational smokefree parks policy:

*What we are doing as a community is putting a line in the sand and saying what we believe is appropriate. ... it is inappropriate to smoke around our young people*.'[67]

## Discussion

### The types of arguments used

Overall, interviewees drew on two main discourses in their discussion of possible government regulation of smoking in public and private spaces, a 'rights' and a 'child protection' argument. The primary discourse was about adults' rights to conduct themselves as they wished in private spaces. Smoking was seen by some of the interviewees as a personal affair, and if conducted in private spaces, its consequences for children were in practice secondary to a smoker's right to smoke.

However, while homes were accepted by all as private (and by nearly all as inviolate regarding smoking), there was disagreement about cars. Some interviewees considered them to be private (for smoking); others saw them as places into which government regulation of smoking could reasonably extend. Going further into the public domain, we found even more varied opinions about smokefree public parks and playgrounds. Some interviewees had reservations about *regulating *'personal' behaviour; and some local councillors were opposed to *any *council smokefree policy, educational or regulatory. Other interviewees argued that smokefree parks were justified, though often only if introduced in a 'bottom-up' fashion generated by community concern.

The other main (counter) argument or discourse presented by some interviewees involved the need for child protection, for children to be free from exposure to SHS, and from smoker role-modelling. The 'rights of children argument' had enormous discursive value for many interviewees, who assumed that they did not need to explain or justify the protection of children. This assumption may reflect a 'normalising discourse'[68] - an increasing prevalence of assumptions around such protection.

### The possible rationales for policymakers

There are a number of possible rationales underlying the policymakers' views. First, a preference for the rights of adults or smokers, over the rights of children. As in other research on smokefree policymaking, ideology was a strong driver of policy, in this case the ideology of individual (adult or voter) rights, rather than community and child rights [36,37,69].

Such preference is contrary to international law, and to many ethical systems [70-72]. There are a number of means by which this preference could be addressed, including increasing the voice of children. As Eekelaar (1992) stated, 'No society will have begun to perceive its children as right-holders until adults' attitudes and social structures are seriously adjusted towards making it possible for children to express views, and towards addressing them with respect' [73]. Survey and qualitative data on child attitudes and SHS exposure may be a start towards achieving this [74].

Secondly, the policymakers may have been reflecting a general unease with government intervention into the personal and private. In New Zealand, as in other Western countries, the home is often seen as 'the place ... where the individual can exert autonomy away from the coercive gaze of the employer and the state. It is the private realm in an increasingly intrusive world' [75]^p.311^.

Those tensions could have been particularly sensitive for policymakers in the period of interviews, which overlapped with the run-up to the October 2008 New Zealand general parliamentary election. There was a strong theme in the political discourse at the time of the intrusion of the state into private activities, particularly due to new (2007) restrictions on adults punishing children physically [76,77]. This concern with political difficulties illustrates the potential gap between public support for change as demonstrated by surveys,[57-59] and the *perceptions *by policymakers of the public mood (ie, the political discourse). Alternately, even if policymakers knew of the support for change, they may have perceived difficulties in translating public support into policy decisions that could be defended.

Thirdly, the literature that emphasises the barriers of effectiveness, cost and implementation issues was confirmed by the strong concern about enforcement issues. The lack of awareness of smokefree car legislation in other jurisdictions compounded these concerns.

Fourthly, there may have been a perception by the policymakers that smoking is an activity which does not *sufficiently *impact on others to justify regulation, particularly in private spaces. So while child death or physical injury from violence or car crashes could be seen as warranting legislation in New Zealand and elsewhere,[2,78,79] the interviewees did not appear to acknowledge or appreciate the 'private' deaths and injury from SHS as warranting regulatory intervention. A contrast can be made between the physical abuse of children (in which the harm to the child is usually immediate, clearly causative, and often involves identifiable individuals) with SHS harm. SHS harm is generally delayed, and is most clear at the population level (eg, statistics for child respiratory hospitalisations).

Similarly, policymakers may also not perceive of the *example *of adult smoking as sufficiently immediate or serious enough to require regulation. Furthermore, they may not understand the extent to which nicotine addiction reduces smokers' 'choices', thereby blunting smokers' consideration of the effects on children. Finally, there is the general issue of 'sticky norms'. When an activity is seen as 'normal' by many, policymakers can tend to resist change [80].

### What is public, or of public concern?

The findings are consistent with much of the literature on the public/private divide, [11-14] that suggest that the perceptions of the divide are fluid, contested, and with disparate policy needs and consequences. For smokefree interventions to protect children, the divide may be very different compared to interventions for child or adult physical injury, for annoyance to the public (eg, from loud music from homes), or for other legal 'nuisances' [43,81-83].

### Implications for public health action

If policymakers do not consider childrens' exposure to SHS to be a serious health issue, one grave enough to require interventions, one option is to effectively inform them of the scientific evidence regarding its harmfulness. They may also need to be better informed of public support for interventions, and of the success of interventions (eg, smokefree car laws) elsewhere. As mentioned above, providing more powerful and effective voices for childrens' views may also help the political forces for childrens' rights.

The success of efforts to regulate smoking around children may depend on the political climate [32,44]. This was supported by the finding that some interviewees suggested that efforts to reduce children's exposure to smoking in private places should be aimed at re-constructing the culture around smoking, creating new norms in which any smoking around children would be considered unacceptable. Such use of cultural norms (eg, promoted via mass media campaigns) has the ethical as well as the political advantage of not directly threatening a smoker's autonomy [84].

So what are other options besides smokefree regulations? The population-level options for limiting or preventing smoking around children include strategies to reduce the overall smoking prevalence in society. More specific strategies around SHS include education and social marketing, comprehensive smoking cessation support services (particularly in pregnancy and for parents), and measures to promote the overall reduction of smoking prevalence [20]. The monitoring of the prevalence of SHS-in-homes, and of the consequences (eg, child hospitalisations) could help provide a base for advocacy and government action [33]. Thus, for instance, particular help for families to quit smoking or to make their homes smokefree could be required where a child has doctor-diagnosed asthma or another chronic respiratory condition.

### Strengths, limitations and further research

A major strength of this study is the obtaining of in-depth interview information from a relatively large number of policymakers.

A possible limitation is that due to the overlap with the election period, the interviewee's opinions may have been skewed away from supporting regulations by the contemporaneous discourse around state intrusion. Other limitations may arise from the recruitment of interviewees (uneven left-right political balance), the lower recruitment of politicians compared to officials, and possible social desirability bias in the answers from all interviewees. However, we found a similar spread of opinions across both politicians and officials, and from both 'left' and 'right' politicians.

The aim of the research may have an inherent bias, in that we assume that some regulation to protect children is necessary. However, the data collection and interpretation focused on the range of views on public/private/personal boundaries, rather than adopting stances on these boundaries.

Although the findings come from one country with relatively advanced tobacco control policies in some areas, the policymaker's views on smokefree options may be common to a number of other developed countries. New Zealand society privileges many features of individualism and minimal roles for government, which make some of these findings reasonably germane to other Anglo-American countries [85]. Concern with the regulation of what may be seen as private behaviour,[86-88] is also common in at least Anglo-American countries, and there are similar gaps between public support for change and policies to reduce smoking around children [21,22]. However, the findings may be less relevant to countries where the state is perceived as having a strong protective role, eg, some European/Scandinavian countries [89,90].

Further research on policymaker opinions about what is public and private in a broad range of countries may illuminate directions for child-friendly policies. Similarly, research is needed on why 'the protection of vulnerable children' discourses are politically effective in some Australian states,[32] and possibly in other places with smokefree car laws, but not in similar jurisdictions. Other avenues of research could investigate the way interviewees seemed to assume that legislation should extend into *some *'private spaces' but shouldn't in others. In such research interviewers could pose this issue (using examples) to interviewees who did not bring up these different approaches themselves. Research could also explore further the awareness of policymakers about surveyed public opinion on smokefree issues.

## Conclusions

We found a general disjunction between policymakers' wish to protect children, and their opinions on ensuring that this protection occurred in perceived 'private' spheres. Policymaker reservations about smokefree regulations arose from both ideological considerations and perceived practicalities. If societies wish to ensure protection of children from SHS they may need to increase the voice of children and youth, provide better information to policymakers, and promote mass media campaigns to denormalise smoking in the presence of children.

## Abbreviations

DHB: District Health Boards; MP: Member of Parliament; SHS: secondhand smoke.

## Competing interests

Although we do not consider it a competing interest, for transparency, the authors (except for GR) have undertaken work for health sector agencies working in tobacco control.

## Authors' contributions

GR carried out the analysis of the data, wrote the first text drafts, and participated in all subsequent drafts. GT conceived, designed and supervised the study, helped gather data and analyse it, and participated in all text drafts. NW helped design the study, and participated in all text drafts, SH helped design the study, gather data, and analyse it; and participated in all text drafts. RE was a significant interpreter of data, and participated in all text drafts. HG helped design the study, supervised an arm of it, helped analyse data, and participated in all text drafts. TL helped gather data and analyse it, and participated in all text drafts. All authors read and approved the final manuscript.

## Pre-publication history

The pre-publication history for this paper can be accessed here:

http://www.biomedcentral.com/1471-2458/10/797/prepub
